# Ruthenium-quercetin coordinated nanotherapeutics with macrophage polarization regulation to rapidly promote bacterial-infected wound healing

**DOI:** 10.1016/j.mtbio.2025.101983

**Published:** 2025-06-20

**Authors:** Zhongxiong Fan, Guoyu Xia, Fukai Zhu, Nan Yang, Aixia Ma, Yanrong Shi, Ziwen Jiang, Xianhui Zhou, Zhenqing Hou

**Affiliations:** a School of Pharmaceutical Sciences, Institute of Materia Medica, Xinjiang University, Urumqi, 830017, China; b Xinjiang Key Laboratory of Biological Resources and Genetic Engineering, College of Life Science and Technology, Xinjiang University, Urumqi, 830017, China; c Department of Gynecology, Beijing Obstetrics and Gynecology Hospital, Capital Medical University, Beijing Maternal and Child Health Care Hospital, Beijing, 100006, China; d Department of Cardiac Pacing and Electrophysiology, The First Affiliated Hospital of Xinjiang Medical University, Urumqi, 830054, China; e Xinjiang Key Laboratory of Cardiac Electrophysiology and Remodeling, The First Affiliated Hospital of Xinjiang Medical University, Urumqi, 830054, China; f College of Materials, Xiamen University, Xiamen, 361005, China

**Keywords:** Biomimetic materials, Ruthenium, Quercetin, Macrophage polarization, Synergistic antibacterial

## Abstract

The hypoxic and inflammatory microenvironment induced by bacterial invasion of wound tissue severely hinders the healing process. In recent years, photothermal therapy (PTT) based on metal–polyphenol coordination networks has garnered considerable attention due to its potent antibacterial effects, biofilm-disrupting capabilities, and intrinsic enzyme-like activities. In this study, a metal–polyphenol coordinated nanotherapeutic system was constructed via synergistic coordination-driven self-assembly between the transition metal ruthenium and quercetin (referred to as QRs). These QRs demonstrated excellent photothermal conversion efficiency and antibacterial performance, while also catalyzing the decomposition of hydrogen peroxide into oxygen under inflammatory conditions. In an *in vitro* LPS-stimulated hypoxic macrophage model, treatment with QRs significantly reduced intracellular reactive oxygen species (ROS) levels and alleviated hypoxia, thereby downregulating hypoxia-inducible factor-1α (HIF-1α) expression and promoting macrophage polarization from the proinflammatory M1 phenotype to the anti-inflammatory M2 phenotype. In a murine bacterial wound infection model, QRs effectively suppressed the expression of inflammatory cytokines, including tumor necrosis factor-α (TNF-α) and interleukin-6 (IL-6), while enhancing endothelial cell proliferation and increasing the proportion of M2 macrophages at the wound site. These changes collectively facilitated the timely transition of the wound into the remodeling phase and maximized the regenerative potential of endogenous immune cells. Overall, this work highlights the potential of ruthenium-based materials for photothermal antibacterial therapy and proposes a promising strategy to remodel the wound microenvironment for enhanced tissue repair.

## Graphical abstract

**Figure:**
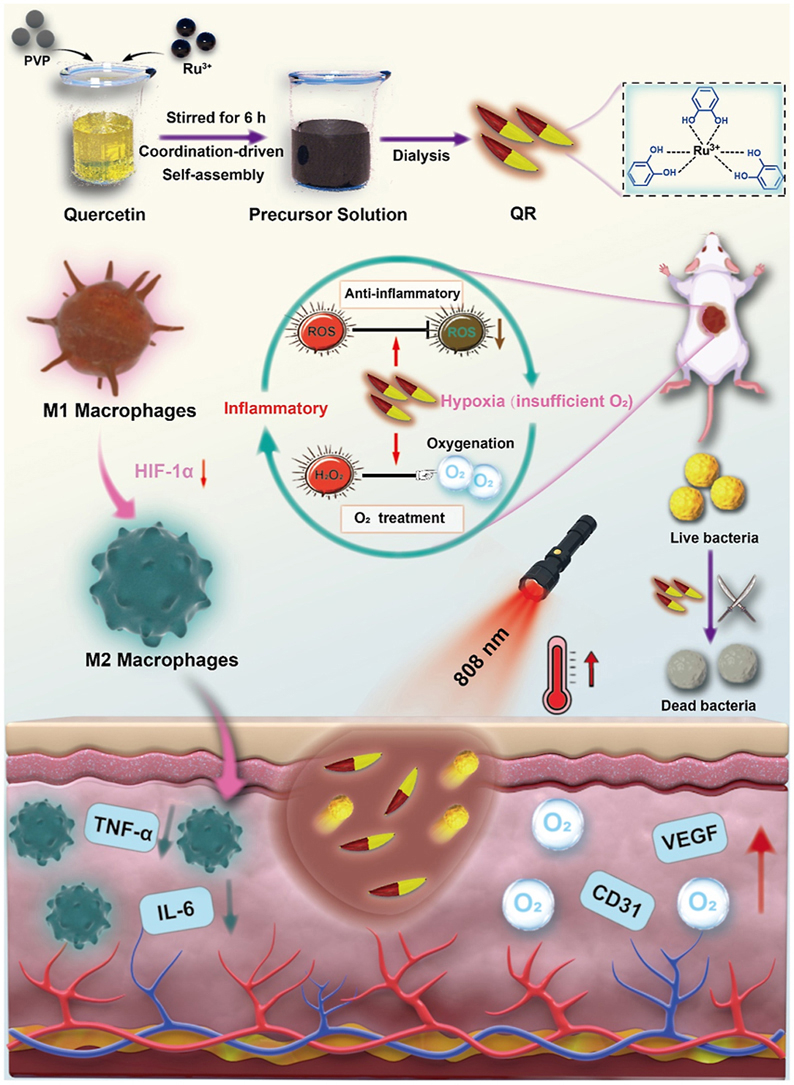

## 1 Introduction

Bacterial invasion of wound tissue often results in extensive tissue damage, sustained chronic inflammation, and delayed healing, thereby imposing substantial physiological and economic burdens on patients [[1], [2], [3]]. Although antibiotics remain the mainstay of clinical treatment for bacterial infections, the formation of bacterial biofilms within wounds presents a critical therapeutic challenge [[4], [5], [6]]. These biofilms act as physical and biochemical barriers, markedly enhancing bacterial resistance to antibiotics—by factors ranging from 100 to 1000—while simultaneously disrupting immune clearance mechanisms and impairing tissue repair [7,8]. Therefore, there is an urgent need for therapeutic strategies capable of disrupting biofilms and effectively eradicating bacteria.

PTT has emerged as a promising noninvasive treatment modality for bacterial infections [9,10]. Through the introduction of photothermal agents, PTT enables the conversion of light energy into heat, facilitating the disruption of biofilms and compromising bacterial structures such as proteins and membranes [11,12]. Additionally, localized hyperthermia has been reported to stimulate vasodilation, enhance blood perfusion, and promote the release of epidermal growth factor and VEGF, thereby supporting angiogenesis and accelerating wound healing [[13], [14], [15]]. Currently, Various metal-based nanomaterials, including Ru NPs, Au NPs, Ag NPs, CuS, and MoS_2_, have been extensively investigated for their photothermal applications [[16], [17], [18], [19]]. Among these, ruthenium (Ru), a low-cost precious metal, stands out due to its abundant d-electron orbitals and active valence electron layer, exhibiting CAT activity, glutathione peroxidase activity, and peroxidase activity [[20], [21], [22]]. Thus, Ru-based materials have garnered attention in the biomedical field. However, studies on Ru-based materials in photothermal antibacterial therapy remain limited.

Notably, the enzyme-like activity of Ru—particularly its CAT-like function—has shown considerable potential in ameliorating the hypoxic conditions within wound tissues [[23], [24], [25]]. By catalyzing the decomposition of excessive H_2_O_2_ in the inflammatory microenvironment, Ru enables in situ oxygen generation, which downregulates HIF-1α expression and promotes the phenotypic transition of pro-inflammatory M1 macrophages toward the reparative M2 phenotype [26]. This process accelerates tissue repair and enhances wound healing. Accordingly, the integration of Ru into antibacterial therapeutic platforms holds promise for improving overall treatment outcomes. Nevertheless, persistent bacterial proliferation and the associated exacerbation of local inflammation often cause wounds to remain in the inflammatory phase, ultimately resulting in chronic, non-healing lesions [[27], [28], [29]]. To address this challenge, the application of antioxidant agents capable of modulating the inflammatory microenvironment is urgently required to facilitate the transition toward tissue regeneration.

Quercetin is a natural flavonoid compound primarily found in plants and fruits [30,31]. It exhibits significant properties such as ROS scavenging, anti-inflammatory, antibacterial, and anticancer effects [32]. As a potent antioxidant, quercetin can neutralize free radicals and reduce the release of pro-inflammatory cytokines, such as tumor necrosis factor-alpha (TNF-α), interleukin-6 (IL-6), and interleukin-1β (IL-1β), by inhibiting key signaling pathways, including nuclear factor-kappa B (NF-κB) and mitogen-activated protein kinase (MAPK) in immune cells [33,34]. Furthermore, studies have shown that the antibacterial mechanism of quercetin primarily induces bacterial cell death through the disruption of the cell wall, cell membrane, and nucleic acids [35]. In light of these properties, the rational design of a nanoplatform integrating antioxidant, antibacterial, photothermal, and CAT-like functionalities is of significant importance. Such a multifunctional platform could synergistically modulate the inflammatory microenvironment, promote tissue repair, and thereby enhance the overall therapeutic efficacy [36].

**Fig. 1:**
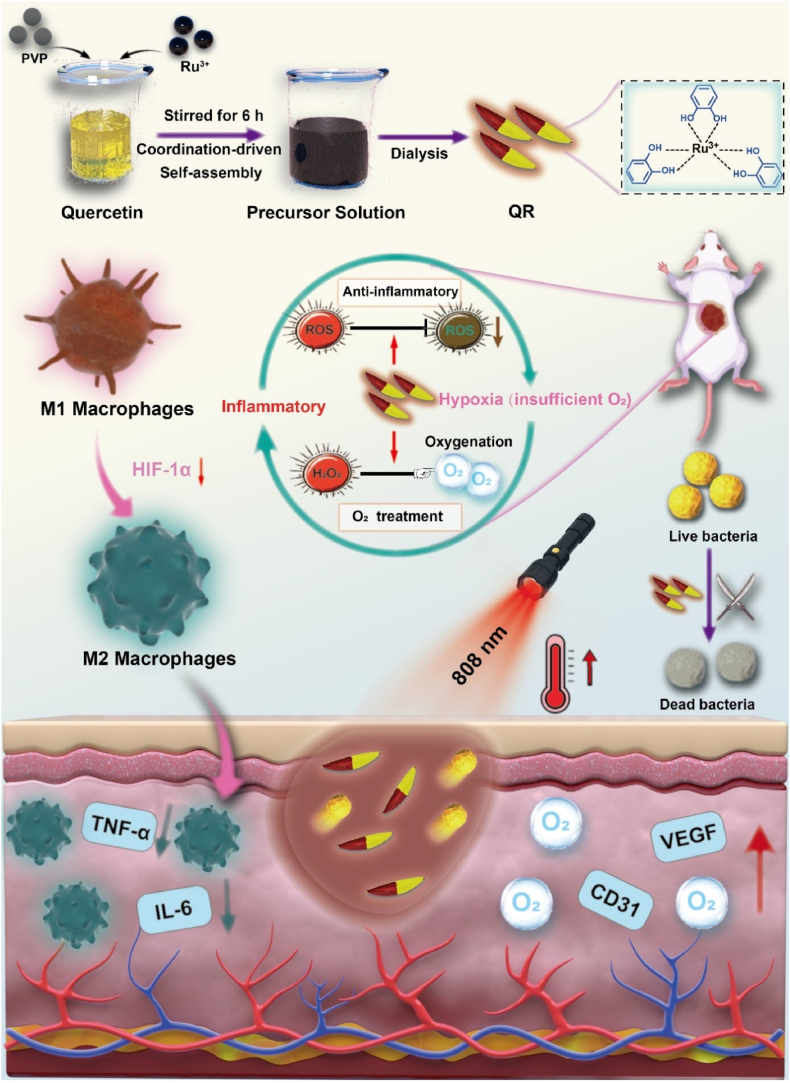
Schematic illustration of the preparation of QRs and therapeutic strategy for accelerating wound healing following bacterial invasion. QRs promote the wound healing process via multiple mechanisms: (1) photothermal sterilization to eliminate bacteria; (2) enhanced macrophage polarization; (3) modulation of inflammatory cytokine release; and (4) promotion of angiogenesis facilitated by O_2_ generated from CAT enzymatic activity.

In this study, we developed ruthenium-quercetin nanotherapeutics through a coordination-driven self-assembly approach, which integrates photothermal properties, catalytic activity, antibacterial effects, and antioxidant capabilities to facilitate rapid wound healing following bacterial invasion (Fig. 1). This metal–polyphenol coordination platform confers several notable advantages. **First**, the synthesis process is green and straightforward, enabling the modular integration of multifunctionality through the controlled adjustment of the metal-to-polyphenol ratio. **Second**, a synergistic antibacterial effect is achieved, whereby Ru facilitates photothermal-induced bacterial eradication owing to its strong near-infrared absorption, while quercetin contributes additional antibacterial activity, together resulting in effective elimination of methicillin-resistant *Staphylococcus aureus* (MRSA). **Third**, both *in vitro* and in vivo evaluations indicate that QRs substantially suppress intracellular ROS levels, inhibit the secretion of pro-inflammatory cytokines such as TNF-α and IL-6, and accelerate tissue regeneration. **Fourth**, the CAT-like activity of QRs catalyzes the conversion of H_2_O_2_ to O_2_, thereby alleviating local hypoxia, reducing HIF-1α expression, and promoting macrophage polarization from the M1 to the M2 phenotype. Collectively, this multifunctional nanotherapeutic system provides a promising strategy for modulating the inflammatory and hypoxic microenvironments of infected wounds, offering new insights into the design of advanced antibacterial platforms for efficient tissue repair.

## 2 Results and discussion

### 2.1 Characterization and coordination mechanism of QR

**Fig. 2:**
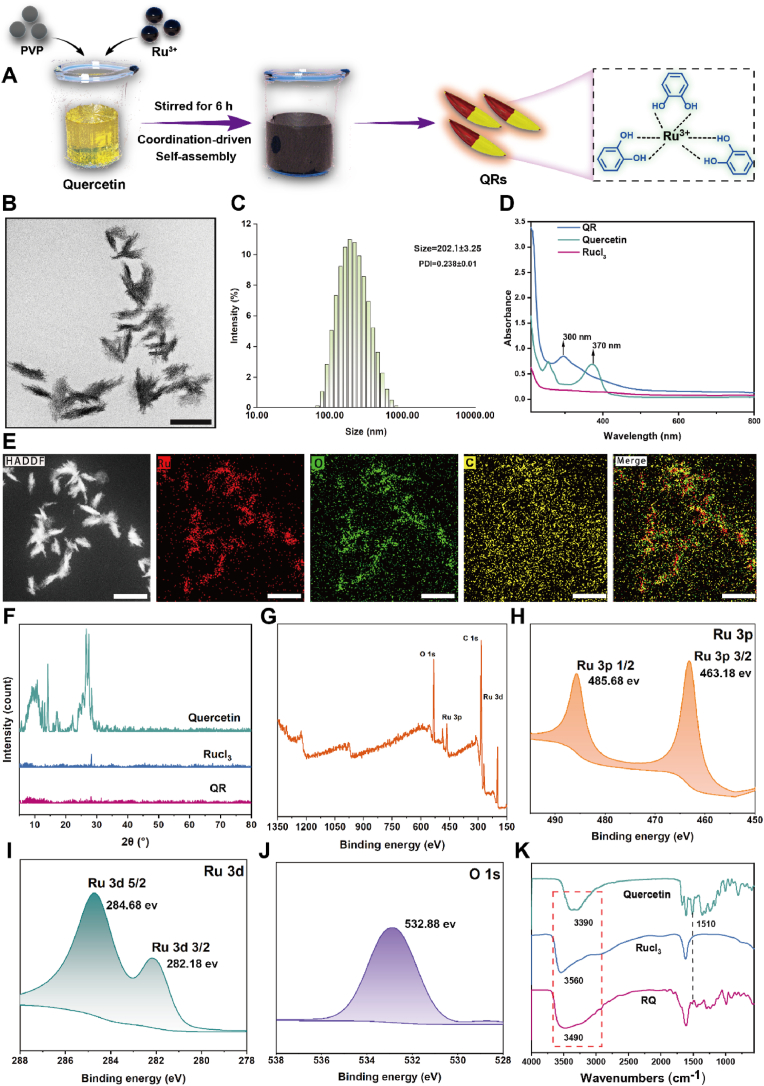
(A) Schematic illustration of the preparation process for QR. (B) TEM image of QR, scale bar = 300 nm. (C) Hydrodynamic diameter and polydispersity index of QR measured by DLS. (D) UV–Vis–NIR absorption spectra of quercetin, RuCl_3_, and QR. (E) HAADF-TEM image and EDS elemental mapping of QR, scale bar = 300 nm. (F) XRD spectra of quercetin, RuCl_3_, and QR. (G) XPS wide scan spectra of QR. (H) Ru 3p, (I) Ru 3d, and (J) O 1s high-resolution spectra of QR. (K) FTIR spectra of quercetin, RuCl_3_, and QR.

Benefiting from the intrinsic antibacterial capabilities of metal–phenolic networks, QRs were fabricated through a quercetin-mediated noncovalent assembly strategy. To preliminarily assess the formation of metal–phenolic coordination structures, solutions containing varying molar ratios of quercetin and ruthenium trichloride were prepared, and their Tyndall effect was subsequently examined. As shown in Fig. S1, a distinct Tyndall effect was observed at a molar ratio of 1:4, and the corresponding solution remained clear and transparent, indicating the formation of colloidal particles. This phenomenon also implied a notable improvement in the aqueous solubility of quercetin. Furthermore, analysis of particle size distributions at different molar ratios revealed a gradual decrease in particle diameter with increasing ruthenium content. At a 1:4 ratio, the QRs exhibited the smallest average particle size, approximately 202 nm. This size reduction may be attributed to the coordination-driven self-assembly between ruthenium and quercetin, which facilitated a more compact and ordered molecular organization, thereby promoting the formation of uniform nanoscale composite structures. Therefore, subsequent experiments were conducted using the optimized 1:4 M ratio. As shown in Fig. 2A, QRs were synthesized in methanol using a co-solvent system combined with dialysis, resulting in the formation of metal–polyphenol nanosheets. Transmission electron microscopy (TEM) images reveal that QR exhibits a leaf-like nanosheet structure (Fig. 2B). To further investigate the structural characteristics, high-magnification TEM imaging was performed. As shown in Fig. S2, the absence of distinct lattice fringes indicated the formation of a metal–amorphous coordination structure, which is generally associated with improved dispersibility and enhanced catalytic activity. Morphological analysis further showed that the nanoleaves had an average length of approximately 198 nm and a width of 40 nm, features that may contribute to their colloidal stability and antibacterial performance (Fig. S2). Dynamic light scattering (DLS) analysis confirmed a hydrodynamic diameter of approximately 202 nm and a polydispersity index (PDI) of 0.238, indicating uniform particle distribution and good dispersibility (Fig. 2C). To assess the physiological stability and potential drug release behavior of QRs, changes in particle size and PDI were monitored over a five-day period. Only minimal fluctuations were observed, suggesting excellent colloidal stability, likely due to the robust ionic coordination environment. In addition, when exposed to H_2_O_2_ stimulation, QRs exhibited markedly accelerated drug release, indicating a favorable responsiveness to oxidative microenvironments and potential for enhanced localized therapeutic delivery (Fig. S3)

Given the observable color change during the synthesis process, the UV–vis absorption spectrum of QRs was analyzed to investigate potential electronic transitions. As seen in Fig. 2D, the characteristic absorption peak of free quercetin at 370 nm exhibited a blue shift to 300 nm upon complex formation. This shift is likely attributable to the coordination between Ru^3+^ and the phenolic hydroxyl groups of quercetin, which may induce a redistribution of electron density and alter the π–π transition energy levels, resulting in absorption at a lower wavelength. Scanning transmission electron microscopy and elemental mapping confirmed the uniform distribution of Ru, C, and O within the QR nanosheet matrix (Fig. 2E S4). X-ray diffraction (XRD) analysis further revealed changes in crystallinity. Whereas quercetin displayed sharp diffraction peaks indicative of a high degree of crystallinity, these peaks became obscured and were replaced by broad, diffuse signals following coordination with Ru^3+^ (Fig. 2F). This transition suggests the formation of a disordered metal–amorphous composite structure, possibly resulting from coordination-induced molecular rearrangement.

To verify the elemental composition and coordination environment, X-ray photoelectron spectroscopy was performed. Consistent with energy-dispersive spectroscopy results, signals corresponding to Ru, C, and O were clearly identified (Fig. 2G). High-resolution spectra revealed that the Ru 3d_5_/_2_ and 3d_3_/_2_ peaks were located at 284.68 eV and 282.18 eV, respectively, while the Ru 3p_1_/_2_ and 3p_3_/_2_ peaks were detected at 485.68 eV and 463.18 eV (Fig. 2H–I). Additionally, the O 1s peak appeared at 532.88 eV (Fig. 2J). These findings suggest that coordination with quercetin may induce partial reduction of Ru^3+^ to lower valence states (Ru^2+^ or Ru), while simultaneously influencing the local electronic environment of oxygen atoms.

To further elucidate the coordination mechanism, Fourier-transform infrared spectroscopy was employed (Fig. 2K). A shift in the hydroxyl stretching vibration band from 3390 cm^−1^ to 3490 cm^−1^ was observed, along with the disappearance of the characteristic 1510 cm^−1^ peak. These spectral changes imply that Ru^3+^ coordinated with hydroxyl groups on the quercetin backbone, affecting both O–H stretching and aromatic ring vibrations.

### 2.2 Photothermal performance, CAT-like activity, and antioxidant capacity of QRs

**Fig. 3:**
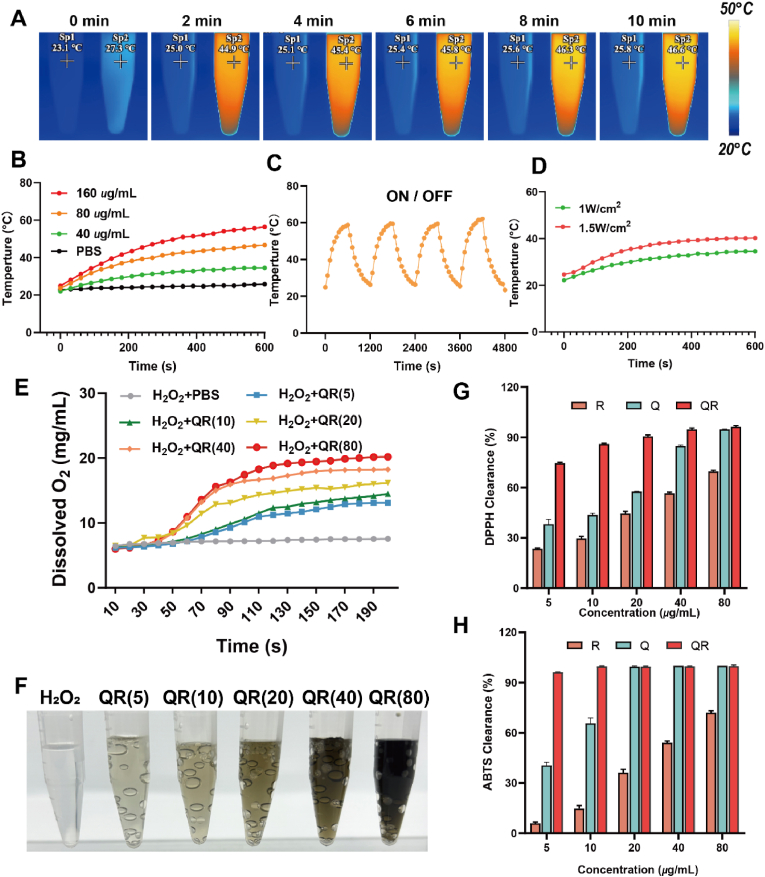
(A) Infrared thermal imaging of QR nanoparticle suspension under near-infrared light (808 nm, 1 W/cm^2^), with PBS on the left and QR (80 μg/mL) on the right. (B) Heating curves of QR nanoparticle suspension at different concentrations under 808 nm, 1 W/cm^2^. (C) Heating and cooling curves of QR nanoparticle suspension over 4 cycles (160 μg/mL, 1 W/cm^2^). (D) Heating curves of QR nanoparticle suspension at different power intensities (40 μg/mL). (E) Oxygen generation from QR at different concentrations in reaction with H_2_O_2_, and (F) corresponding visual images. (G) DPPH radical scavenging activity at different concentrations. (H) ABTS radical scavenging activity at different concentrations. (R: RuCl_3_, Q: quercetin).

To assess the photothermal properties of QRs, solutions at varying concentrations (40, 80, and 160 μg/mL) were irradiated with an 808 nm laser at a constant power of 1 W/cm^2^ for 10 min. As shown in Fig. 3A–B, the temperature of QR solutions increased progressively with time, while the phosphate-buffered saline (PBS) group showed negligible temperature variation under the same conditions. To evaluate photothermal stability, four consecutive heating–cooling cycles were conducted. The maximum temperature during each cycle remained consistent, and cooling curves showed stable behavior, indicating that QRs possess excellent photothermal stability (Fig. 3C). Furthermore, the effect of laser power on thermal response was examined by varying the irradiation intensity, where a power-dependent increase in temperature was observed (Fig. 3D). Based on these data, the photothermal conversion efficiency of QRs was calculated to be 33.8 %, surpassing that of conventional CuS nanoparticles (30.8 %) [37]. Additionally, the molar extinction coefficient was determined to be 0.02787 L mol^−1^ cm^−1^ (Fig. S5). These findings collectively suggest that QRs are well-suited for use in photothermal antibacterial applications requiring both efficient light-to-heat conversion and high structural stability.

Given that bacterial infections often induce a hypoxic and oxidative wound microenvironment, the ability of QRs to relieve hypoxia via catalytic decomposition of H_2_O_2_ was further explored. Hypoxia in wound sites typically arises from impaired perfusion, tissue injury, or prolonged inflammation, and is often accompanied by H_2_O_2_ accumulation, which disrupts normal tissue regeneration [23,38,39]. IIn this context, the CAT-like activity of QRs was examined. As shown in Fig. 3E–F, increasing QR concentrations led to progressively higher oxygen generation, with visible gas bubble formation, indicating efficient catalytic decomposition of H_2_O_2_ and confirming robust CAT-like enzymatic activity.

Furthermore, considering the inflammatory and oxidative nature of infected wounds, the antioxidant capacity of QRs was evaluated using DPPH and ABTS assays. As shown in Fig. 3G–H, a concentration-dependent scavenging of free radicals was observed. Specifically, the DPPH solution shifted from purple to yellow, and ABTS from green to colorless, reflecting active neutralization of reactive species. Notably, at equivalent concentrations, QRs demonstrated superior antioxidant performance compared to controls, highlighting their potential for mitigating oxidative stress in inflamed wound environments.

### 2.3 Evaluation of *in vitro* antimicrobial activity

**Fig. 4:**
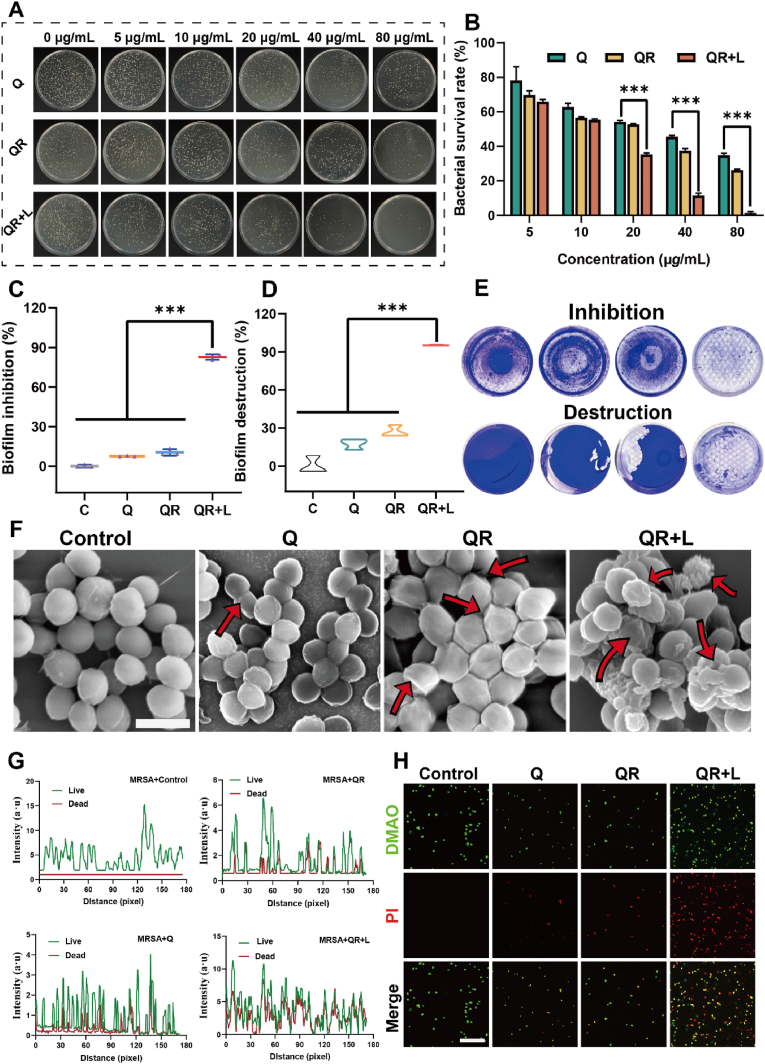
(A) Optical images showing bacterial colony growth after different treatments. (B) Survival rates of MRSA after different treatments. (C) Inhibition rate of MRSA biofilm formation after different treatments, and (D) disruption rate of MRSA biofilm, along with (E) corresponding optical images. (F) Representative SEM images of MRSA after different treatments, scale bar = 1 μm. (G) Co-localization analysis and (H) fluorescence images of dead (red) and live (green) MRSA cells after different treatments, scale bar = 50 μm. (Q: quercetin). Data are presented as mean±SD, *n* = 3. ∗*P* < 0.05, ∗∗*P* < 0.01, ∗∗∗*P* < 0.001. (For interpretation of the references to color in this figure legend, the reader is referred to the Web version of this article.)

Driven by the excellent *in vitro* performance of QRs, we further explored their antimicrobial ability, Initially, QRs at various concentrations were applied to bacterial cultures. As shown in Fig. 4A–B, all treatment groups exhibited a concentration-dependent inhibition of bacterial growth. Notably, the QR + L group demonstrated the most pronounced bactericidal effect, which is likely attributable to the synergistic interaction between the inherent antibacterial activity of quercetin and the photothermal properties of ruthenium under laser irradiation.

Given that biofilm formation constitutes a major barrier to effective infection control and wound healing, the anti-biofilm activity of QRs was subsequently evaluated. A biofilm model was established and subjected to treatment with PBS, Q, QR, and QR + L, followed by crystal violet staining. As shown in Fig. 4C–E, a substantial reduction in the stained area was observed in the QR + L group, indicating a strong ability to both inhibit biofilm formation and disrupt existing biofilms. This effect is presumably mediated by localized hyperthermia, which induces denaturation of membrane proteins and disintegration of the extracellular matrix. To further elucidate the antibacterial mechanism, the morphological changes of methicillin-resistant *Staphylococcus aureus* (MRSA) were examined by scanning electron microscopy (SEM). Bacteria in the PBS group retained their native morphology with smooth surfaces. In contrast, cells in the Q and QR groups exhibited varying degrees of structural deformation, with the QR + L group displaying extensive membrane rupture and cell fragmentation, indicative of severe damage likely caused by membrane destabilization and thermal stress (Fig. 4F). The bactericidal efficacy of QRs was further validated using a live/dead fluorescence staining assay. Green fluorescence (DMAO) marked viable bacteria, while red fluorescence (PI) indicated compromised cells. As illustrated in Fig. 4G and H, the PBS group exhibited exclusively green fluorescence, indicative of viable bacteria. The Q group displayed red fluorescence, signifying bacterial death, but showed minimal co-localization with green fluorescence. In contrast, the QR group demonstrated enhanced co-localization of green and red fluorescence compared to the Q group, which can be attributed to the potent antibacterial activity of quercetin, facilitated by its nanostructured form and improved bioavailability. Importantly, in the QR + L group, a clear co-localization of green and red fluorescence was demonstrated, implying that the QR + L was the strongest in killing bacteria. These results demonstrate the excellent antimicrobial performance and biofilm removal ability of QRs in the presence of laser radiation, which lays the foundation for our next in vivo antimicrobial experiments.

### 2.4 *In vitro* macrophage hypoxia mitigation and inflammation clearance

**Fig. 5:**
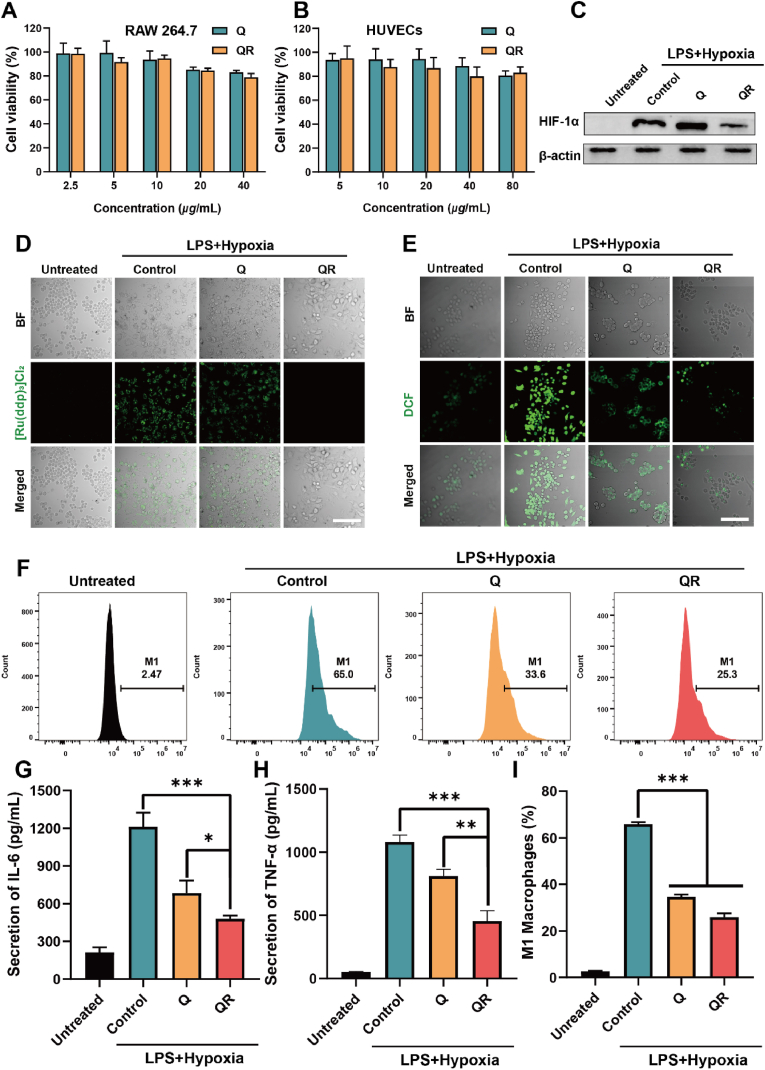
(A) Survival rates of RAW264.7 cells co-cultured with different groups for 24 h. (B) Survival rates of HUVECs co-cultured with different groups for 24 h. (C) Western blot analysis of HIF-1α expression. (D) Representative CLSM images of the oxygen indicator [Ru(dpp)_3_]Cl_2_ in RAW264.7 cells under different treatments. (E) Representative CLSM images of ROS levels in RAW264.7 cells after different treatments. (F) Flow cytometry analysis of the proportion of M1 macrophages. (G) IL-6 secretion levels in RAW264.7 cells after different treatments. (H) TNF-α secretion levels in RAW264.7 cells after different treatments. (I) Semi-quantitative analysis of the proportion of M1 macrophages from F panel. Data are presented as mean±SD, *n* = 3. ∗*P* < 0.05, ∗∗*P* < 0.01, ∗∗∗*P* < 0.001.

Good biocompatibility is essential for subsequent experiments. The MTT assay was employed to assess the cytotoxicity of QR toward RAW264.7 and HUVEC cells, and the results indicated cell viability greater than 70 %, indicating acceptable cytocompatibility for further biological evaluation (Fig. 5A–B). To mimic the inflammatory and hypoxic microenvironment characteristic of infected wounds [[40], [41], [42]], a LPS-induced macrophage model under hypoxic conditions was established. HIF-1α is a key marker for cellular expression under hypoxic conditions. Therefore, we investigated the expression of HIF-1α under different treatments (Fig. 5C). The results showed that QRs significantly reduced the expression of HIF-1α, which may be attributed to the excellent CAT enzyme activity of QRs, which catalyze the generation of O_2_ from H_2_O_2_ in cells, thereby reducing HIF-1α expression. To further validate oxygen generation, the intracellular oxygen level was monitored using the hypoxia-sensitive probe [Ru(dpp)_3_]Cl_2_. Strong fluorescence indicative of hypoxia was detected in the control group, whereas negligible signal was observed following QR treatment, further supporting the oxygen-generating capacity of the material (Fig. 5D). Reactive oxygen species (ROS) levels were subsequently evaluated using the DCFH-DA fluorescent probe. As shown in Fig. 5E, both Q and QR groups markedly reduced ROS accumulation in hypoxic macrophages, with the QR group exhibiting superior scavenging capacity, likely due to the antioxidant effect of quercetin. Given the established link between hypoxia, inflammation, and macrophage polarization, the immunomodulatory potential of QRs was examined. Under LPS and hypoxic stimulation, QR pre-treatment significantly reduced the proportion of pro-inflammatory M1 macrophages, enhanced the polarization toward anti-inflammatory M2 phenotypes, and suppressed the expression of TNF-α and IL-6 (Fig. 5F–I and S6). These effects are presumably mediated by the antioxidant and oxygen-generating properties of QRs, which collectively contribute to the resolution of inflammation and support tissue repair.

### 2.5 *In vivo* antibacterial experiment and wound healing mechanism

**Fig. 6:**
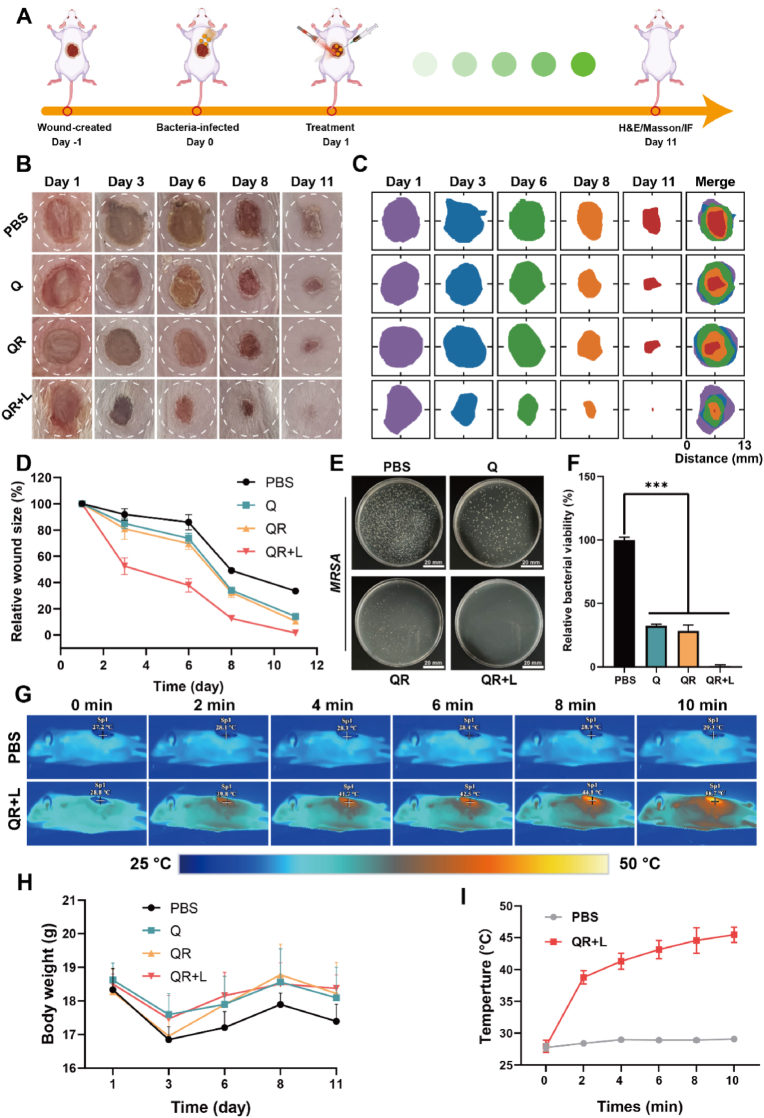
(A) Schematic illustration of the mouse bacterial infection wound model and treatment process. (B, C) Optical images and wound heat maps showing wound healing changes at days 1, 3, 6, 8, and 11 for different treatment groups. (D) Quantitative analysis of wound size. (E) Bacterial colonies in wound tissue after different treatments. (F) Quantitative analysis of bacterial colonies. (G) Infrared thermal imaging of mice from the PBS and experimental groups (808 nm, 1 W/cm^2^, 10 min). (H) Body weight changes of mice throughout the entire treatment period. (I) Quantitative analysis of infrared thermal imaging of mice. Data are presented as mean±SD, *n* = 3. ∗*P* < 0.05, ∗∗*P* < 0.01, ∗∗∗*P* < 0.001.

Owing to the excellent photothermal performance, CAT-like activity, and antioxidant properties of QRs, an in vivo mouse model of MRSA infection was established to evaluate their antibacterial efficacy and wound healing potential. As shown in Fig. 6A, MRSA-infected wounds were successfully generated, followed by treatment with PBS, Q, QR, or QR combined with laser irradiation (QR + L). The progression of wound healing was monitored over 11 days. As illustrated in Fig. 6B–D, all treatment groups facilitated wound repair to varying degrees compared to the PBS control group, with the QR + L group exhibiting the most pronounced healing effect. Notably, nearly complete wound closure was achieved in the QR + L group by Day 11, along with effective elimination of biofilm.

To directly assess the antibacterial performance, wound tissue samples were collected post-treatment for bacterial colony quantification. As shown in Fig. 6E–F, bacterial load was significantly reduced in all treatment groups, with the QR + L group displaying the fewest colonies, correlating with its superior wound healing outcome. Infrared thermal imaging confirmed localized temperature elevation at the wound site in the QR + L group, which is consistent with its photothermal bactericidal effect (Fig. 6G–I). Moreover, no significant body weight changes were observed in any group throughout the treatment period, indicating favorable in vivo biocompatibility.

**Fig. 7:**
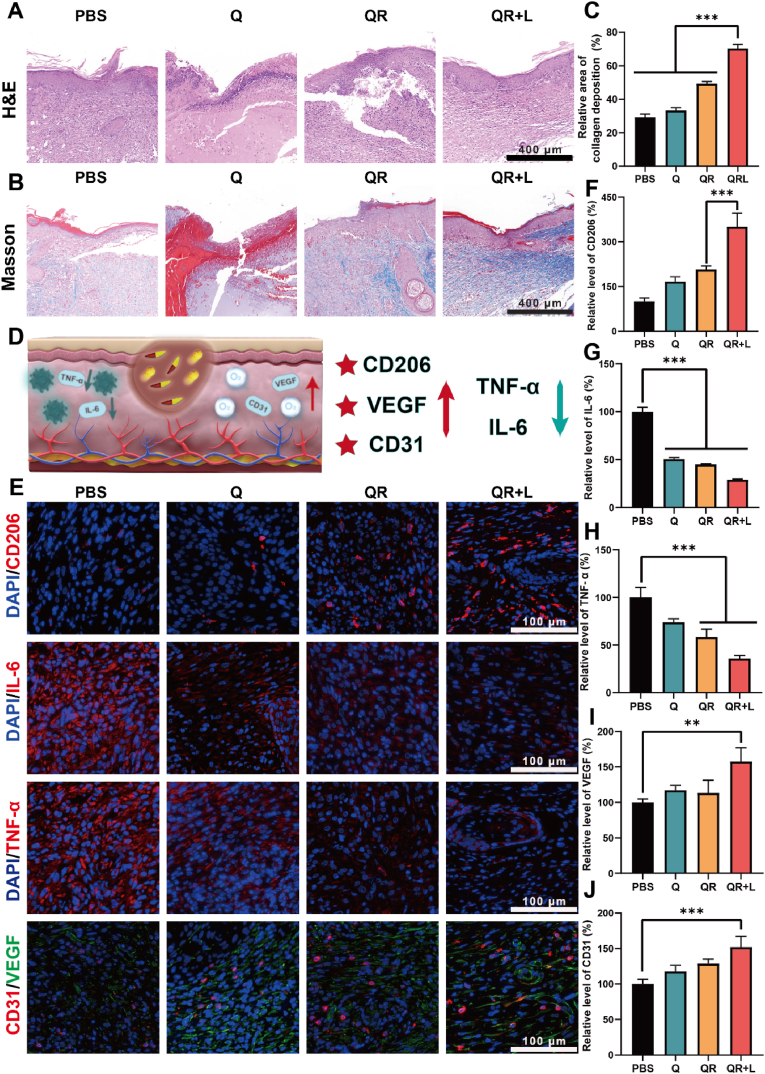
(A) H&E staining of wound tissue at the end of the entire treatment period. (B) Masson trichrome histological staining of wound tissue. (C) Semi-quantitative analysis of collagen fibers in wound tissue. (D) Schematic representation of reduced inflammatory factors and increased angiogenic factors in wound tissue. (E) Immunofluorescence staining of wound tissue for CD206, IL-6, TNF-α, CD31, and VEGF after different treatments. (F) Semi-quantitative analysis of CD206 expression. (G) Semi-quantitative analysis of IL-6 expression. (H) Semi-quantitative analysis of TNF-α expression. (I) Semi-quantitative analysis of VEGF expression. (J) Semi-quantitative analysis of CD31 expression. Data are presented as mean±SD, *n* = 3. ∗*P* < 0.05, ∗∗*P* < 0.01, ∗∗∗*P* < 0.001.

To further investigate the mechanisms underlying tissue regeneration, histological and immunofluorescence analyses were performed on harvested wound tissue, including H&E, Masson, CD206, TNF-α, IL-6, CD31, and VEGF staining. As shown in Fig. 7A, the epidermal thickness in the QR + L group was significantly increased, with a notable difference observed compared to the PBS group, which was consistent with the trend of epithelial wound healing, which may be attributed to the anti-inflammatory properties and oxygen-generating capacity of the treatment, thereby promoting the proliferation and repair of epithelial cells. These results suggest that the treatment effectively facilitated epithelial repair at the wound site, thereby accelerating the overall wound healing process. As shown in Fig. 7B–C, collagen fibers (blue area) were significantly increased in the QR + L group compared to the other groups, indicating that the QR + L group may have promoted collagen deposition and tissue repair.

Inflammatory and angiogenic factors in wound tissue are important indicators for assessing wound healing (Fig. 7D). In order to assess the immune response of the wound tissue, immunofluorescence staining for CD206,TNF-α, and IL-6 was performed, and the results showed that the control group had a very small percentage of M2-type macrophages and was accompanied by a severe inflammatory response (large amount of red fluorescence for TNF-α, and IL-6), whereas the QR + L group showed a higher amount of red fluorescence labeled by CD206, and the red fluorescence for TNF-α, IL-6 red fluorescence was attenuated (Fig. 7E). Relative semi-quantitative analysis of the results showed that the QR + L group had the largest proportion of M2-type macrophages and secreted significantly lower levels of TNF-α, IL-6 inflammatory factors relative to the PBS group (Fig. 7E–H). In addition, the endothelial cell marker CD31 and pro-angiogenic growth factor VEGF indicate angiogenesis activity, supporting tissue repair and regeneration. As shown in the figure (Fig. 7E), the red fluorescence and green fluorescence areas of CD31 and VEGF in the QR + L group were significantly higher than those in other groups, and the relative quantitative analysis also confirmed the same result that the expression of CD31 and VEGF was increased in the QR + L group. This may be attributed to the unique photothermal effects and catalytic activity of QRs, which effectively improve the local oxygenation environment in the wound area, further enhancing oxygen concentration and ameliorating local oxygenation status (Fig. 7I–J). Together, the above findings suggest that the QR + L group improved the wound healing process by modulating the immune response and promoting tissue repair.

**Fig. 8:**
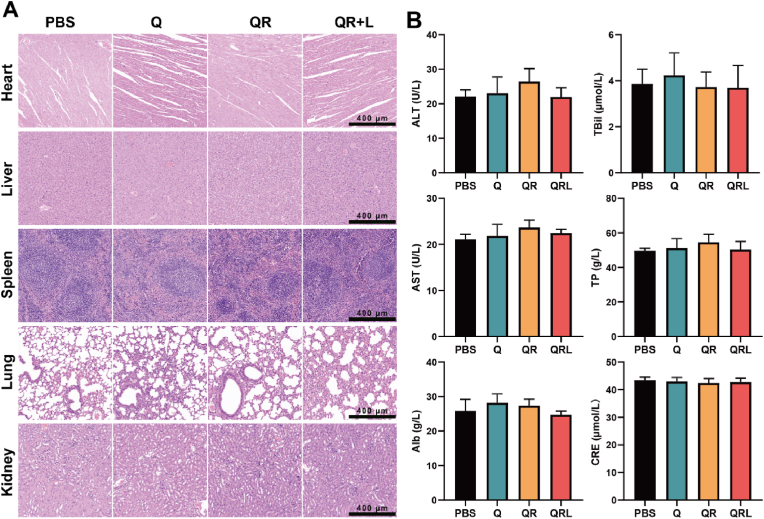
(A) H&E staining of heart, liver, spleen, lung, and kidney tissues. (B) Serum biochemical markers at the end of the 11-day treatment period. Data are presented as mean±SD, *n* = 3.

In addition, H&E staining and blood biochemical analysis of major organs (heart, liver, spleen, lungs, and kidneys) in mice at the end of the treatment revealed no significant inflammatory responses or tissue damage, and all biochemical parameters remained within normal ranges (Fig. 8A–B). Furthermore, hemolysis assays confirmed that the hemolysis rates were all below 5 %, indicating that QRs possess a certain degree of biosafety (Fig. S7).

## 3 Conclusions

Overall, we have developed a nanotherapeutic formulation based on a metal-polyphenol coordination network strategy, which integrates photothermal, anti-inflammatory, and catalytic properties to promote rapid wound healing following bacterial invasion. This formulation achieves large-scale bacterial eradication through the synergistic effects of photothermal therapy and the antibacterial activity of quercetin, while also inhibiting and disrupting biofilm formation by MRSA. *In vitro* LPS + hypoxic macrophage model, QRs significantly reduced intracellular ROS levels and increased intracellular O_2_ supply, thereby reducing HIF-1α expression and further promoting the polarization of M1 macrophages to M2 macrophages. Notably, in vivo bacterial infection model, QRs increased the proportion of M2 macrophages, stimulated endothelial cell proliferation, reduced the secretion of TNF-α and IL-6 in the tissue microenvironment, and accelerated the rapid healing of wound tissue. In conclusion, the nanoplatform constructed using the ruthenium-quercetin coordination network strategy demonstrates efficient antibacterial performance, along with the ability to manage M2 macrophage polarization and alleviate the inflammatory microenvironment, making it an effective approach to accelerate tissue repair.

## 4 Experimental methods

### 4.1 Materials

Ruthenium(III) chloride hydrate (RuCl_3_·xH_2_O), quercetin, and glutaraldehyde were purchased from Shanghai Macklin Biochemical Technology Co., Ltd. Polyvinylpyrrolidone (PVP) was obtained from Shanghai Aladdin Biochemical Technology Co., Ltd. Crystal violet staining solution, ROS probes, and bacterial live/dead staining kits were provided by Shanghai Biotime Biotechnology Co., Ltd. [Ru(dpp)_3_]Cl_2_ oxygen sensing probe was obtained from Shanghai Mocon Biotech Co., Ltd. Elisa kits for IL-6 and TNF-α were purchased from Elabscience. CD206, IL-6, TNF-α, CD31, and VEGF antibodies were all supplied by Wuhan Servicebio Technology Co., Ltd.

### 4.2 Synthesis of QR

Quercetin (12 mg) was accurately weighed and dissolved in 5 mL of methanol to obtain solution A. Ruthenium (III) chloride hydrate (48 mg) was dissolved in 5 mL of methanol to prepare solution B. Polyvinylpyrrolidone (PVP, 160 mg) was dissolved in 5 mL of deionized water to form solution C. All solutions were sonicated for 10 min to ensure complete dissolution. Solution C was slowly added dropwise to solution An under stirring, until the solution became a transparent pale yellow. Then, solution B was added dropwise to the mixture, resulting in a color change from pale yellow to black. The reaction mixture was stirred for 6 h to ensure complete reaction. Subsequently, the reaction mixture was dialyzed for 12 h using a dialysis membrane with a molecular weight cutoff of 1000 Da to remove free drugs and residual methanol. The final product was stored at 4 °C in following experiment.

### 4.3 Instrument

The nano sizes of QR were measured using a Malvern Laser Particle Size Zeta Potential Analyzer (Zetasizer Nano ZS90, UK). The morphology of QR was observed using Transmission Electron Microscopy (TEM, JEOL JEM 2100F). Fourier Transform Infrared Spectroscopy (FTIR, VERTEX 70, Germany), X-ray Photoelectron Spectroscopy (XPS, Escalab 250XI, Germany), and X-ray Diffraction (XRD, D8 advance, Germany) were employed to acquire the corresponding spectral data. The ultraviolet absorption and fluorescence values at specific wavelengths were determined using a multifunctional microplate reader (Molecular Devices, SpectraMax Id5, USA). Confocal Laser Scanning Microscopy (Nikon, AIR HD25, Japan) was utilized to obtain CLSM images. Immune outcomes were detected by flow cytometry (Beckman Coulter, CytoFLEX, USA).

### 4.4 Photothermal performance evaluation

1 mL of QR solution at different concentrations was placed in 1.5 mL centrifuge tubes and irradiated with an 808 nm laser (1.0 W/cm^2^) for 10 min. Temperature changes were recorded using an infrared thermal imaging camera. At a concentration of 40 μg/mL, samples were irradiated with lasers of different power densities for 10 min, and temperature changes were recorded. Additionally, to evaluate the stability of QR, four photothermal cycling experiments were performed.

### 4.5 *In vitro* CAT enzyme activity assay

500 μM/mL H_2_O_2_ was added to 1 mL centrifuge tubes, followed by the addition of QR solutions at different concentrations. Oxygen consumption was measured and recorded using a dissolved oxygen meter over a period of 210 s, and the experimental process was photographed.

### 4.6 *In vitro* antioxidant activity evaluation

QR solutions at different concentrations were added to DPPH and ABTS solutions to evaluate their antioxidant properties. The absorbance at 517 nm and 734 nm was measured using a microplate reader, and the antioxidant scavenging rates were calculated.

### 4.7 Antibacterial assay

MRSA bacterial suspension in the logarithmic growth phase was diluted to 1 × 10^6^ CFU/mL (OD = 0.1) with sterile medium. 500 μL of bacterial suspension was incubated with 500 μL of drug solution from different groups for 4 h. The samples were then irradiated with an 808 nm laser (1 W/cm^2^) for 10 min. The treated bacterial suspension was diluted 10^4^ times, and 100 μL of the sample was plated on an agar plate. The plate was spread evenly with a glass rod and incubated at 37 °C for 24 h. Colony counts were recorded and photographed.

### 4.8 Biofilm destruction experiment

100 μL of MRSA bacterial suspension (1 × 10^8^ CFU/mL) was added to a 24-well plate, followed by the addition of 2 mL of LB medium containing 2 % glucose. The plate was incubated at 37 °C for 48 h to form a complete biofilm. Subsequently, the biofilm was treated with Q, QR, and QR + L for 4 h. The QR + L group was irradiated with an 808 nm laser (1 W/cm^2^) for 10 min, followed by incubation at 37 °C for 24 h. After discarding the supernatant, the biofilm was washed three times with PBS, stained with crystal violet for 30 min, and washed again three times with PBS. The plate was then air-dried. The absorbance of the solubilized crystal violet in ethanol was measured at 570 nm using a microplate reader, and the biofilm disruption rate was calculated.

### 4.9 Biofilm inhibition experiment

The biofilm inhibition experiment was carried out similarly to the biofilm destruction experiment. The drug solution was mixed with the bacterial suspension and added to a 24-well plate. After 4 h of incubation, the samples were irradiated with an 808 nm laser (1 W/cm^2^) for 10 min, followed by incubation at 37 °C for 24 h. After crystal violet staining, excess dye was washed away, and photographs were taken. The absorbance at 570 nm was measured using a microplate reader.

### 4.10 Bacterial morphological analysis

Bacteria were co-incubated with different materials for 4 h, followed by irradiation with an 808 nm laser (1 W/cm^2^) for 10 min. The treated bacterial samples were fixed overnight in 2.5 % glutaraldehyde at 4 °C. After centrifugation to remove glutaraldehyde, the samples were washed with an ethanol gradient, and the fixed bacteria were collected. A 10 μL aliquot of the bacterial sample was dropped onto a silicon wafer for scanning electron microscopy (SEM) observation.

### 4.11 Bacterial viability and mortality assay

MRSA bacteria were co-incubated with different materials for 4 h, followed by laser irradiation with an 808 nm laser (1 W/cm^2^) for 10 min. The bacteria were then stained according to the instructions of the bacterial viability and mortality kit. After staining, CLSM was used to capture images and observe the co-localization of DMAO (green) and PI (red) to determine the bacterial mortality rate.

### 4.12 MTT cytotoxicity assay

MTT assay was used to evaluate the cell viability of human umbilical vein endothelial cells (HUVECs) and RAW264.7 cells treated with materials at different concentrations. HUVECs were incubated with materials at varying concentrations for 24 h. After discarding the supernatant, MTT solution was added, and the cells were incubated for 4 h. Afterward, DMSO was added and mixed for 5 min. The absorbance at 490 nm was measured using a microplate reader, and the data were recorded.

### 4.13 Cellular oxygen monitoring assay

To assess the intracellular oxygen production levels in M1 macrophages under hypoxic conditions, the oxygen indicator [Ru(dpp)_3_]Cl_2_ was used. A total of 1.5 × 10^5^ macrophages per well were seeded into confocal culture dishes and stimulated with LPS (10 μg/mL) for 12 h. Subsequently, the cells were placed in an anaerobic chamber and treated with hypoxia for 4 h, during which [Ru(dpp)_3_]Cl_2_ was added. The cells were then pre-treated with 20 μg/mL of Q and QR solutions for 6 h. Finally, the fluorescence signal of [Ru(dpp)_3_]Cl_2_ was observed using CLSM (excitation wavelength: 488 nm, emission wavelength: 610 nm).

### 4.14 Intracellular ROS scavenging assay

A total of 2 × 10^5^ RAW264.7 cells were seeded into confocal culture dishes and stimulated with LPS (10 μg/mL) for 12 h. The cells were then subjected to hypoxia treatment for 6 h and pre-treated with 20 μg/mL of Q and QR solutions for 6 h. The intracellular reactive oxygen species levels were detected using the DCFH-DA probe (diluted at 1:3000). Finally, CLSM was used to capture fluorescence images of the cells.

### 4.15 Macrophage polarization and inflammatory cytokine detection

A total of 1.5 × 10^5^ RAW264.7 cells were seeded into 12-well plates and stimulated with LPS (10 μg/mL) for 12 h. Following this, the cells underwent hypoxia treatment for 6 h and were pre-treated with 20 μg/mL of Q and QR solutions for 6 h. The cells were collected and stained with PE-CD80 antibody for 20 min. The expression level of CD86 was then detected using flow cytometry. The supernatants from the 12-well plates after the above treatments were collected, diluted at a ratio of 1:50, and the levels of secreted cytokines were measured using IL-6 and TNF-α ELISA kits.

### 4.16 *In vivo* antibacterial and wound healing assays

Six to eight-week-old BALB/C mice were selected and the hair around the spinal region was shaved. Circular skin wounds of 8 mm in diameter were made on the back of the mice. A 50 μL MRSA bacterial suspension (1 × 10^8^ CFU/mL) was inoculated onto the wound area. Twenty-four hours later, the mice were randomly divided into four groups: 1) PBS group, 2) Q group, 3) QR group, 4) QR + L group (Q: 80 μg/mL). During administration, we applied 50 μL of the drug solution and covered the area with a sterile patch to ensure the local concentration of the drug. During the treatment period, the body weight of the mice was monitored, and the wound area was photographed to track changes. After the treatment ended, skin tissues from the wound areas were collected for histological analysis, including HE and Masson staining, as well as immunofluorescence staining to investigate the wound healing mechanism. Finally, routine blood analysis was conducted on the mice.

### 4.17 Statistical analysis

Data were analyzed using Graph Pad Prism (Version 8.0), and data were expressed as the mean ± SD of at least three repeated measurements. t-tests (two-sided)) were performed to analyze the data. *p* < 0.05 was considered statistically significant. ∗*p* < 0.05, ∗∗*p* < 0.01, and ∗∗∗*p* < 0.001.

## CRediT authorship contribution statement

**Zhongxiong Fan:** Writing – original draft, Supervision, Funding acquisition, Data curation, Conceptualization. **Guoyu Xia:** Writing – original draft, Conceptualization. **Fukai Zhu:** Software. **Nan Yang:** Data curation. **Aixia Ma:** Formal analysis. **Yanrong Shi:** Software. **Ziwen Jiang:** Data curation. **Xianhui Zhou:** Supervision, Funding acquisition. **Zhenqing Hou:** Writing – original draft, Supervision.

## Ethics approval and consent to participate

All animal experiments were conducted in accordance with the guidelines of the Ethics Committee of Xinjiang University, and the experiments were approved by the Ethics Committee of Xinjiang University.

## Declaration of competing interest

The authors declare that they have no known competing financial interests or personal relationships that could have appeared to influence the work reported in this paper.

## Data Availability

The authors do not have permission to share data.
