# Supplementary material for: Ruthenium-quercetin coordinated nanotherapeutics with macrophage polarization regulation to rapidly promote bacterial-infected wound healing
Source: Mater Today Bio. 2025 Jun 20;33:101983. doi: 10.1016/j.mtbio.2025.101983 (PMC12226065; doi:10.1016/j.mtbio.2025.101983)
Supplement: Multimedia component 1 [file mmc1.docx]

***Supplementary Material***

**Ruthenium-quercetin coordinated nanotherapeutics with macrophage polarization regulation to rapidly promote bacterial-infected wound healing**

Zhongxiong Fan ^a,1,^*
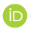
, Guoyu Xia ^a,b,1^, Fukai Zhu ^a,b,1^, Nan Yang ^a,b^, Aixia Ma ^a,b^, Yanrong Shi ^a,b^, Ziwen Jiang ^c , *^, Xianhui Zhou ^d, e, *^, Zhenqing Hou ^a,d,^*
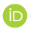
,

^a^ School of Pharmaceutical Sciences, Institute of Materia Medica, Xinjiang University, Urumqi 830017, China

^b^ Xinjiang Key Laboratory of Biological Resources and Genetic Engineering, College of Life Science and Technology, Xinjiang University, Urumqi 830017, China

^c^ Department of Gynecology, Beijing Obstetrics and Gynecology Hospital, Capital Medical University, Beijing Maternal and Child Health Care Hospital, Beijing 100006, China

^d^ College of Materials, Xiamen University, Xiamen 361005, China

^e^ Xinjiang Key Laboratory of Cardiac Electrophysiology and Remodeling, The First Affiliated Hospital of Xinjiang Medical University, Urumqi, 830054, China

^f^ College of Materials, Xiamen University, Xiamen 361005, China

^1^ Zhongxiong Fan, Guoyu Xia, and Fukai Zhu contributed equally to this work

- *Corresponding authors: Dr. Zhongxiong Fan, E-mail: fanzhongxiong@xju.edu.cn; Dr. Ziwen Jiang, E-mail: jiangziwen@ccmu.edu.cn; Dr. Xianhui Zhou, Zhouxhuiyf@xjmu.edu.cn; Dr. Zhenqing Hou, E-mail: houzhenqing@xmu.edu.cn
- **ORCID**

Zhongxiong Fan: https://orcid.org/0000-0001-5751-9530

Zhenqing Hou: https://orcid.org/0000-0002-5537-075X

***Supplementary data***


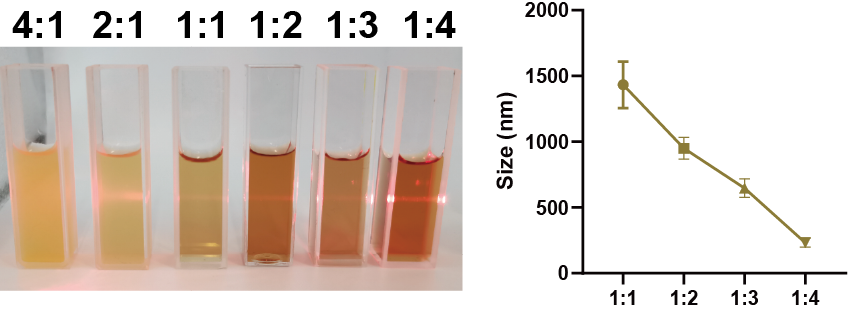


**Figure S1**. Tyndall effect and particle size of ruthenium and quercetin at different molar ratios.


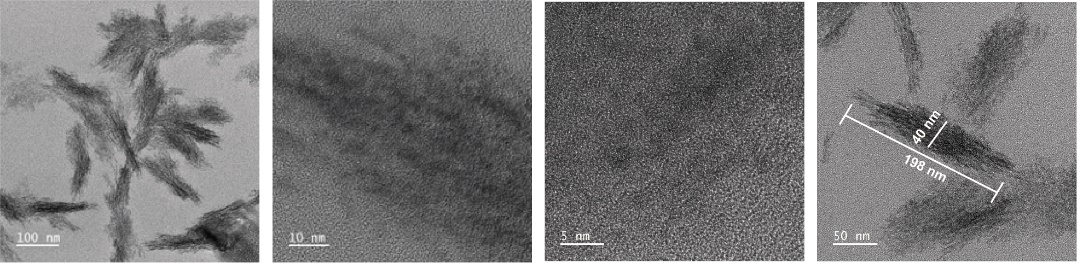


**Figure S2**. High-resolution TEM image of QR.


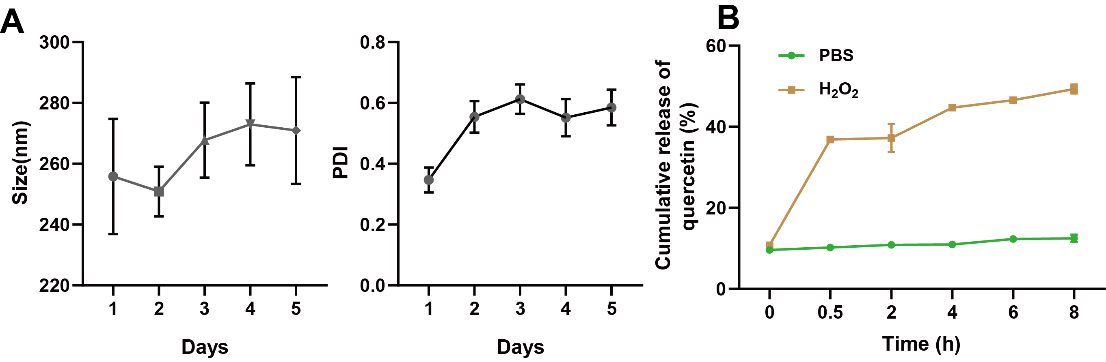


Figure S3. (A) Stability of particle size and PDI of QR over 5 days. (B) Cumulative release of quercetin under H₂O₂ conditions (n=3).
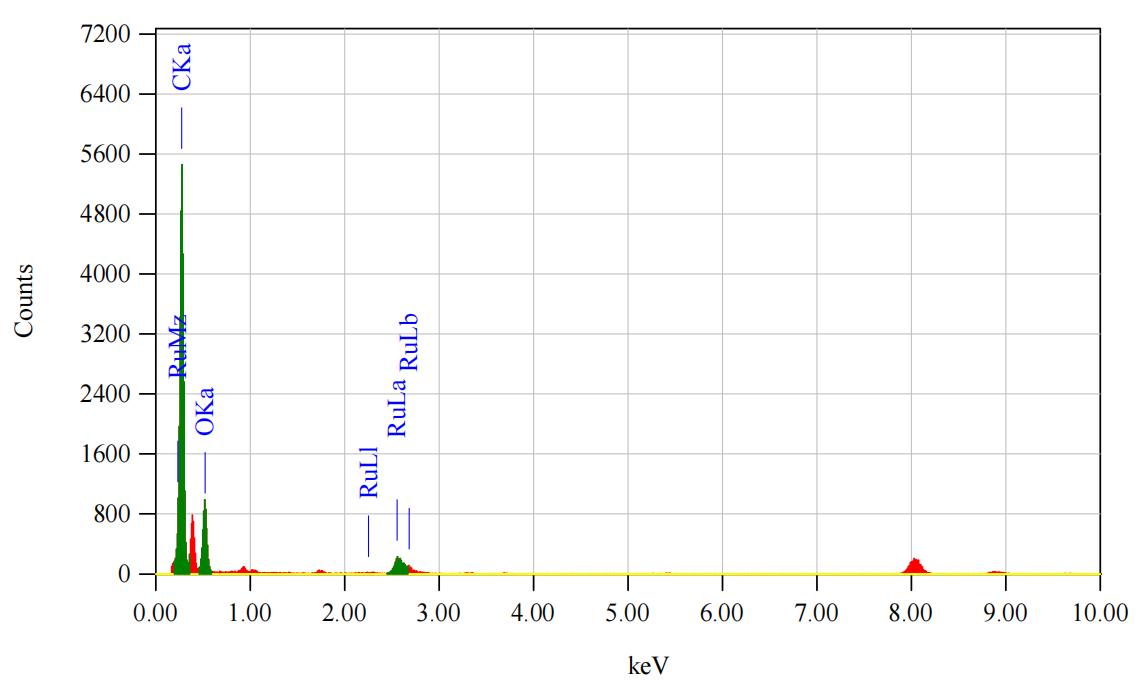


| **Element** | **(keV)** | **Mass%** | **Counts** | **Sigma** | **Atom%** | **Compound Mass%** |
| --- | --- | --- | --- | --- | --- | --- |
| C K (Ref.) | 0.277 | 86.09 | 26402.77 | 0.41 | 92.08 | 1.0000 |
| O K*! | 0.525 | 9.11 | 5467.88 | 0.15 | 7.32 | 0.5110 |
| Ru L* | 2.558 | 4.80 | 1574.27 | 0.17 | 0.61 | 0.9344 |
| **Total** |  | 100.00 |  |  | 100.00 |  |

**Figure S4.** The EDS analysis results of QR.


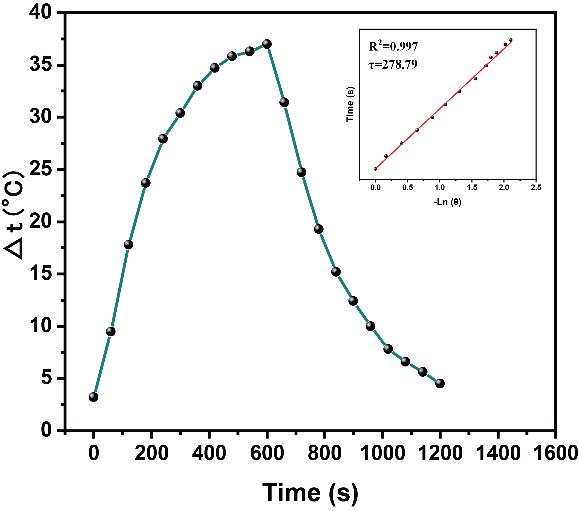


Figure S5. The photothermal conversion efficiency of QR.


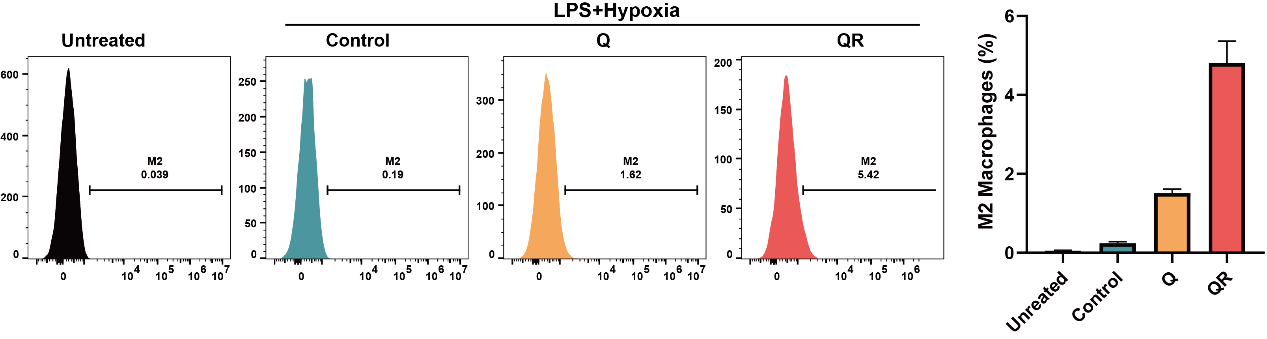


**Figure S6.** The proportion of M2 macrophages after different treatments (*n*=3)


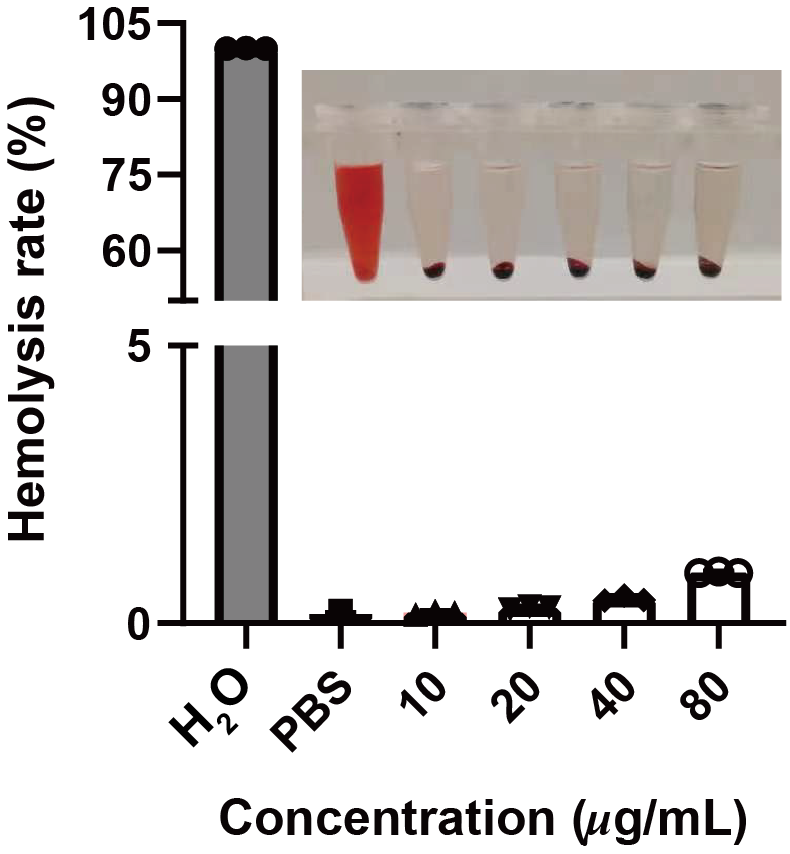


**Figure S7.** Hemolysis rates of water, PBS, QR. Inset: Optical images showing dispersion of water, PBS, QR(10), QR(20) ,QR(40) ,QR(80) in red blood cells. Data are presented as mean ± standard deviation (*n* = 3).
